# An Audit of Whether Patients on the General Adult Inpatient Wards and the PICU in Mersey Care NHS Foundation Trust Who Are Prescribed High Dose Antipsychotic Therapy Are Being Monitored as per Trust Policy

**DOI:** 10.1192/bjo.2024.580

**Published:** 2024-08-01

**Authors:** Declan Hyland, Roopa Singh, Kerry Dainton

**Affiliations:** Mersey Care NHS Foundation Trust, Liverpool, United Kingdom

## Abstract

**Aims:**

High Dose Antipsychotic Therapy (HDAT) should only be used in exceptional circumstances, as there is little evidence to suggest that higher than recommended doses of antipsychotics are more clinically effective than standard doses, with potential side effects being greater. In practice, there are several clinical scenarios where HDAT may be prescribed and the potential benefits must outweigh the potential risks. NICE guidelines for psychosis and schizophrenia advise that dosages outside the range given in the British National Formulary should be justified and recorded.

This audit aimed to determine whether patients on the 16 general adult inpatient wards and Psychiatric Intensive Care Unit (PICU) in Mersey Care NHS Foundation Trust who are prescribed HDAT are managed as recommended by the Trust's HDAT policy.

**Methods:**

A list of all inpatients admitted to the 16 general adult inpatient wards and the PICU in the Trust between 17^th^ and 20^th^ of July 2023 was obtained. The electronic prescription record for each patient was scrutinised to determine whether the patient was prescribed HDAT. For each HDAT patient, the patient's electronic psychiatric record was analysed to determine whether baseline physical health assessments – ECG, BMI, waist circumference, BP, pulse rate, FBC, U and Es, LFTs, serum prolactin level, HbA1c level and random serum total cholesterol level and lipid profile were completed before commencing HDAT. Each HDAT patient was reviewed to determine whether a cardiovascular assessment was completed prior to commencing HDAT.

**Results:**

29 inpatients on the 16 general adult wards and the PICU were prescribed HDAT. The Trust's HDAT policy states 13 baseline physical health assessments should be completed prior to commencing HDAT. For the 29 patients, 13 baseline physical health assessments should have been completed on 378 occasions. On 98 occasions, the patient refused and these were excluded from the compliance. There were 226 (81%) occasions they were completed and 54 (19%) when they weren't completed. 12 (41%) of the 29 HDAT patients had a cardiovascular assessment done prior to commencing HDAT.

**Conclusion:**

There are a significant number of inpatients in whom not all the required baseline physical health assessments prior to commencing HDAT are completed. A cardiovascular assessment is an important aspect of deciding whether to prescribe a patient HDAT and yet commonly not completed. There is a need to ensure that nursing and medical staff on the inpatient wards are aware of the Trust's HDAT policy and need to refer to and to adhere to it.

